# Strong Gametocytocidal Effect of Methylene Blue-Based Combination Therapy against Falciparum Malaria: A Randomised Controlled Trial

**DOI:** 10.1371/journal.pone.0005318

**Published:** 2009-05-05

**Authors:** Boubacar Coulibaly, Augustin Zoungrana, Frank P. Mockenhaupt, R. Heiner Schirmer, Christina Klose, Ulrich Mansmann, Peter E. Meissner, Olaf Müller

**Affiliations:** 1 Centre de Recherche en Santé de Nouna, Nouna, Burkina Faso; 2 Institute of Tropical Medicine and International Health, Charité – University Medicine Berlin, Berlin, Germany; 3 Biochemistry Centre, Ruprecht-Karls-University, Heidelberg, Germany; 4 Institute of Medical Biometrics and Informatics, Medical School, Ruprecht-Karls-University, Heidelberg, Germany; 5 Institute of Bioinformatics and Epidemiology, Medical School, Ludwig Maximilians University München, Germany; 6 Department of Tropical Hygiene and Public Health, Medical School, Ruprecht-Karls-University, Heidelberg, Germany; University of California Los Angeles, United States of America

## Abstract

**Background:**

With the availability of new preventive and curative interventions, global malaria control has been strengthened significantly in recent years. Drugs effective in reducing malaria gametocytaemia might contribute to local elimination and possible long-term eradication. We here report on the effects of methylene blue (MB)-based malaria combination therapy on gametocytaemia during a randomised-controlled trial in Burkina Faso.

**Methods:**

An open-label randomised controlled phase II study in 180 children aged 6–10 years with uncomplicated falciparum malaria was conducted in Nouna, north-western Burkina Faso. Children were randomised to MB–artesunate (AS), MB–amodiaquine (AQ), and AS-AQ (local standard of care). Overall follow-up was for 28 days, follow-up for gametocytaemia was for 14 days.

**Findings:**

The treatment groups were similar in baseline characteristics and there was only one loss to follow-up. Compared to AS-AQ, both MB-containing regimens were associated with significantly reduced gametocyte carrier rates during follow-up days 3, 7, and 14. This effect was seen both in patients with and without *P. falciparum* gametocytaemia at baseline.

**Interpretation:**

MB reveals pronounced gametocytocidal activity which appears to act against both existing and developing *P. falciparum* gametocytes. MB-based combination therapy thus has the potential to reduce transmission of *P. falciparum* malaria in endemic regions, which has important implications for future elimination and eradication strategies.

**Trial Registration:**

ClinicalTrials.gov NCT00354380

## Introduction

Malaria remains the most important parasitic disease globally, and in Sub-Saharan Africa (SSA) in particular [1]. New or rediscovered interventions such as insecticide-treated mosquito nets [2], artemisinin-based combination therapy (ACT) [3], and vaccines [4], together with substantially increased funding are promising in terms of improved malaria control and possibly, eradication [5]–[8]. In fact, as a consequence of a large-scale rollout of available interventions, major reductions in malaria morbidity and, partially, child mortality have recently been observed in areas of Kenya, Tanzania and Ethiopia [9]–[11].

Gametocytocidal antimalarials, and the artemisinines in particular, may accelerate such progress in that they reduce post-treatment transmission [12]–[14]. Yet, artemisinin derivatives do not prevent post-treatment transmission completely [15], and in view of emerging ACT resistance in South-East Asia [16]–[18], alternative drugs effective against gametocytes are urgently needed. Methylene blue (MB), the first synthetic drug ever used against malaria [19], has received renewed attention in recent years [20]. MB is a subversive substrate and specific inhibitor of the *P. falciparum* disulfide reductases and in addition inhibits the parasite's heme detoxification [20], [21]. MB in combination with a number of partner drugs has been shown to be safe and effective in a West-African population with a high prevalence of glucose-6-phosphate dehydrogenase deficiency [22]–[25]. Interestingly, plasmoquine, an antimalarial drug developed as a derivative MB, is known to be highly effective against gametocytes [26]. In this context it is also worth mentioning that genes encoding the putative target proteins of MB [20], [21] were present in the gametocyte-specific cDNA library originally established in Kaslow's laboratory (27). Against this background, we analysed the efficacy of MB-based combination therapy against gametocytes using data from a randomised controlled phase II study in Burkina Faso.

## Methods

The protocol for this trial and supporting CONSORT checklist are available as supporting information; see Checklist S1 and Protocol S1.

The study was conducted in October/November 2006 in the urban research zone of the *Centre de Recherche en Santé de Nouna* (CRSN) in Nouna Health District, north-western Burkina Faso. The area is highly endemic for malaria with most clinical cases occurring during or briefly after the rainy season which lasts from June until October [27]. Despite an ACT policy since 2005, malaria control in the study area continues to be mainly based on home treatment with chloroquine (CQ) [28], [29].

### Study design and objectives

The study was an open label randomized controlled phase II trial on safety and efficacy of MB-artesunate (AS) and MB-amodiaquine (AQ) in children with uncomplicated falciparum malaria, with blinding only for the laboratory technicians involved. The primary objective was to investigate the safety of the combinations MB-AS and MB-AQ in children with uncomplicated falciparum malaria. The secondary objective was to determine the efficacy of these MB-based combinations in the treatment of children with uncomplicated falciparum malaria. The study was designed to have a statistical power of 80% in order to detect a difference in the number of adverse events of at least 20% among study groups that was significant at the five percent level [25].

Post-treatment gametocyte carriage was initially not planned to examine and specified later as an additional secondary endpoint. The methodology of the trial has been published elsewhere [25], and only key aspects are briefly repeated here.

### Study population and procedures

Only children from Nouna town were participating in this study. Inclusion criteria were: age 6–10 years, ability to swallow tablets, uncomplicated falciparum malaria (axillary temperature ≥37.5°C and ≥1,000 *P. falciparum* asexual parasites per µL blood), and written informed consent given by the parents/caretakers. Exclusion criteria were signs of severe malaria, any apparent other disease, and malaria treatment – except CQ – with western drugs and/or antibiotics with antimalarial potency during the preceding week.

180 children were randomly assigned to receive directly observed treatment with either MB-AS, MB-AQ, or AS-AQ. The latter is the official first-line antimalarial in Burkina Faso. MB (Urolene Blue®, Star Pharmaceuticals, USA) was given at a dose of 10 mg per kilogram of body weight twice daily over three days, AS (Artesunate, Guilin Pharmaceuticals Co., Ltd., PR China) at a dose of 4 mg per kilogram of body weight once daily over three days, and AQ (Essential Drug Store, Ministry of Health, Burkina Faso) at a dose of 10 mg per kilogram of body weight once daily over three days. Children with fever ≥38.5°C received a standard dose of 10 mg per kilogram paracetamol tablets every 6 hours (Essential Drug Store, Ministry of Health, Burkina Faso) until symptoms subsided.

Follow-up of study children was for 28 days using a slightly modified version of the latest WHO protocol on antimalarial drug efficacy testing [30]. Children were followed up on days 1, 2, 3, 7, 14, and 28. Mothers and caretakers were encouraged to come back at any time between scheduled visits in case of unforeseen symptoms.

A finger-prick blood sample was taken on days 0, 2, 3, 7, 14, and 28, and during unscheduled visits. From this, malaria parasitaemia and haematocrit values were determined using standard CRSN procedures [31]. Filter paper blood samples served as DNA source for PCR-based differentiation of recrudescences from reinfections [25]. Thick and thin blood films were examined by two experienced laboratory technicians supervised by one of the investigators (BC). Asexual parasites (and later gametocytes during slide re-examination) were counted on thick blood films against 200 white blood cells (WBCs) and parasite density was calculated assuming an average WBC count of 10,000/µL. Slides were declared negative if no parasites were seen in 400 fields on the thick film. For quality control, a 10% random sample of blood films is regularly cross-checked at the Heidelberg School of Tropical Medicine, which has always demonstrated an overall high consistency between the different laboratory technicians with regard to parasite prevalence and density [28]. After initial reading, all study slides were stored under controlled conditions in the laboratory of the CRSN.

In early 2008, all slides from the trial (only day 0 until day 14) were systematically re-examined by two experienced laboratory technicians supervised by one of the investigators (BC) for *P. falciparum* gametocytaemia prevalence and density, both in patients with ACPR and in patients without ACPR. In view of the short half-life of both AS and MB, these drugs would have no effect on new infections emerging after day 14. Thus, gametocytaemia occurring after day 14 was not considered.

### Statistical analysis

The effects of different treatment arms on gametocytaemia were determined using per protocol analysis. The Chi square test (Chi) was used to compare proportions, and the non-parametric Wilcoxon-Mann-Whitney test (WMW) to compare metric or ordinal data. In order to use the closed testing procedure for multiple testing adjustment when comparing three groups, a global test is needed which compares three groups for differences. This is done by an exact chisquare test which is also valid for small sample sizes. When possible, estimates and the corresponding 95% confidence interval are given. Calculations were performed with the program SAS release 9.1 (SAS® Institute Inc, Cary, NC, USA). Multiple testing between single arms in a three-armed trial is handeled by the closed testing procedure: If a difference between all three arms is assessed by a global test on significance level 5%, it is possible to use the same level for the three two group comparisons. This procedure keeps the family wise error rate.

### Ethical aspects

The study protocol was approved by the Ethics Committee of the Medical Faculty at Heidelberg University and the local Ethics Committee of Burkina Faso.

## Results

**Figure 1:**
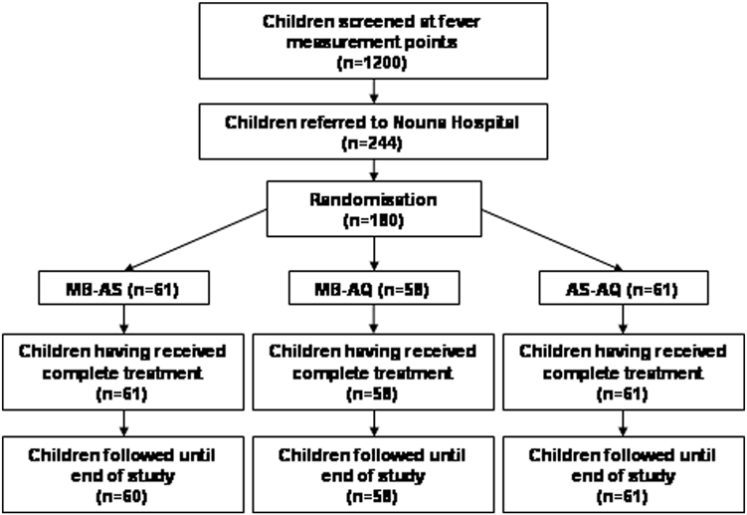
Flow chart of study patients.

One hundred and eighty children (61 MB-AS, 58 MB-AQ, 61 AS-AQ) were included in the study (Figure 1). There was only one loss to follow-up. Patients' demographical, clinical and parasitological characteristics at baseline did not differ between study groups, except for a small difference in weight (p = 0.039; Table 1). In particular, CQ pre-treatment, parasite density, and gametocyte carrier rate did not differ significantly between the treatment groups, although the latter tended to be increased in children receiving AQ-AS (Table 2). Efficacy data of the study drugs have been published elsewhere. Briefly, PCR-corrected rates of adequate clinical and parasitological response at day 28 of follow-up were 62% for MB-AS, 82% for AS-AQ and 95% for MB-AQ [25].

**Table 1:** Characteristics of study children at enrolment.

Characteristic	MB-AS group (n = 61)	MB-AQ group (n = 58)	AS-AQ group (n = 61)
Female sex (%)	30 (49)	32 (55)	22 (36)
Median age in years (range)	7 (6–10)	6 (6–10)	7 (6–10)
Median weight in kg (range)	20 (14–32)*	19 (13–38)*	19 (13–33)*
Median haematocrit in % (range)	34 (26–44)	36 (26–42)	34 (24–40)
Median number of *P. falciparum* trophozoites/µl (range)	33,000 (1,000–265,050)	28,700 (1,000–200,000)	31,000 (1,000–258,000)
Prevalence of *P. falciparum* gametocytes (%)	9 (15)	7 (12)	15 (25)
Median number of *P. falciparum* gametocytes/µl (range)	120 (80–160)	200 (40–320)	80 (40–1,280)
Median duration of the current disease episode in days (range)	2 (1–7)	2 (1–7)	2 (1–7)
Reported CQ treatment of current disease episode (%)	6 (10)	12 (21)	10 (16)

* There was a small but significant difference in weight between the study groups (p = 0.039).

**Table 2:** Efficacy of the study regimens against *P. falciparum* gametocytaemia.

				p-values			
	MB-AS group N = 61	MB-AQ group N = 58	AS-AQ group N = 61	global	MB-AS vs. MB-AQ	MB-AS vs. AS-AQ	MB-AQ vs. AS-AQ
Day 0							
- Prevalence (95% CI)	9 (14.8% [7%; 26%])	7 (12.1% [4%; 20%])	15 (24.6% [14%; 37%])	0.168	0.790	0.255	0.100
- Median density (range)	120.0 (80.0–160.0)	200.0 (40.0–320.0)	80.0 (40.0–1280.0)				
Day 2							
- Prevalence (95% CI)	4 (6.6% [2%; 16%])	7 (12.1% [5%; 23%])	9 (14.8% [7%; 26%])	0.350	0.355	0.240	0.790
- Median density (range)	100.0 (80.0–200.0)	120.0 (80.0–480.0)	160.0 (80.0–1200.0)				
Day 3							
- Prevalence (95% CI)	2 (3.3%, [1%; 11%])	0 [0%; 6%]	9 (14.8% [7%; 26%])	0.002	0.496	0.054	0.003
- Median density (range)	80.0 (40.0–120.0)		160.0 (40.0–440.0)				
Day 7							
- Prevalence (95% CI)	0 [0%; 6%]	0 [0%; 6%]	11 (18.0% [9%; 30%])	<.001		<.001	<.001
- Median density (range)			120.0 (80.0–480.0)				
- missing*	1	1	0				
Day 14							
- Prevalence (95% CI)	0 [0%; 6%]	0 [0%; 6%]	4 (6.8% [2%; 16%])	0.035		0.119	0.110
- Median density (range)			180.0 (160.0–400.0)				
- missing*	3	1	2				

* Patients not available for blood test on respective study days.

The prevalence of gametocytes declined following treatment, regardless of the drug administered. However, both MB-AS and MB-AQ were significantly more effective in this regard than AS-AQ during follow-up (Table 2). No gametoctyes were detected one or two weeks following treatment with the MB-containing regimes whereas their respective prevalences were 18% and 7% in AS-AQ treated children.

Similar findings were observed among patients who exhibited gametocytaemia at enrolment (Table 3).

**Table 3:** *P. falciparum* gametocytaemia during follow-up in patients with *P. falciparum* gametocytaemia on day 0.

				p-values			
	MB-AS group N = 9	MB-AQ group N = 7	AS-AQ group N = 15	global	MB-AS vs. MB-AQ	MB-AS vs. AS-AQ	MB-AQ vs. AS-AQ
Day 2							
- Prevalence (95% CI)	2 (22.2% [3%; 60%])	1 (14.3% [1%; 66%])	8 (53.3% [27%; 79%])	0.142	1.000	0.210	0.165
- Median density (range)	100.0 (80.0–120.0)	480.0 (480.0–480.0)	220.0 (80.0–1200.0)				
Day 3							
- Prevalence (95% CI)	0 [0%; 34%]	0 [0%; 41%]	5 (33.3% [12%; 62%])	0.047		0.118	0.135
- Median density (range)			200.0 (160.0–440.0)				
Day 7							
- Prevalence (95% CI)	0 [0%; 34%]	0 [0%; 41%]	6 (40.0% [16%; 68%])	0.024		0.052	0.121
- Median density (range)			300.0 (80.0–480.0)				
Day 14							
- Prevalence (95% CI)	0 [0%; 34%]	0 [0%; 41%]	4 (26.7% [8%; 55%])	0.134		0.259	0.263
- Median density (range)			180.0 (160.0–400.0)				

Excluding patients with *P. falciparum* gametocytes on day 0 and/or day 2, both MB-based treatment regimes yielded lower *P. falciparum* gametocyte prevalence on day 3 and day 7 compared to AS-AQ (Table 4). On day 14, no gametocytes were detected in all study groups.

**Table 4:** Efficacy of the study drugs against *P. falciparum* gametocytaemia after exclusion of all cases with gametocytes on day 0 and/or day 2.

				p-values			
	MB-AS group N = 50	MB-AQ group N = 45	AS-AQ group N = 45	global	MB-AS vs. MB-AQ	MB-AS vs. AS-AQ	MB-AQ vs. AS-AQ
Day 3							
- Prevalence	1 (2.0% [1%;11%])	0 [0%; 8%]	4 (8.9% [2%; 21%])	0.079	1.000	0.186	0.117
- Median density (range)	120.0 (120.0–120.0)		60.0 (40.0–120.0)				
Day 7							
- Prevalence	0 [0%; 7%]	0 [0%; 8%]	4 (8.9% [2%; 21%])	0.020		0.049	0.117
- Median density (range)			100.0 (80.0–120.0)				
- missing*	1	1	0				
Day 14							
- Prevalence	0 [0%; 8%]	0 [0%; 8%]	0 [0%; 8%]				
- Median density (range)							
- missing*	3	1	2				

* Patients not available for blood test on respective study days.

## Discussion

Treatment of malaria with MB-based regimens not only is more effective than AQ-AS in this part of Burkina Faso [25] but also associated with a markedly reduced prevalence of *P. falciparum* gametocytaemia following treatment. In this first respective study, the gametocytocidal effect of MB-combination treatment significantly exceeded that of AQ-AS. In fact, when following only those subjects who had gametocytaemia at baseline, *P. falciparum* gametocytes were detected exclusively in the ACT control group. In children presenting without gametocytes, the finding of a lower *P. falciparum* gametocytaemia during follow-up in both MB-based combinations remained but was no longer that marked. These results point to a broad activity of MB against both, already existing (older) gametocytes and newly developing (younger) ones. However, due to the limited sample size these observations are based on small and not always statistically significant numbers and need to be interpreted with some caution. Moreover, *P. falciparum* gametocytaemia was not included as a specific endpoint *a priori*. This does not change the reliability of our findings as all slides from the trial were safely stored in the CRSN laboratory and re-examined by blinded laboratory technicians. Measuring the effects of the different treatments on submicroscopic gametocytaemia could have been another option to increase the power of this study. For instance, in a recent trial in Tanzanian children, gametocytes were detected in 23% and 89% by microscopy and real-time PCR assays, respectively [32], and data from The Gambia and Kenya indicate the transmission potential of submicroscopic gametocytaemia [33]. However, standardised collection of appropriate samples to address this issue was not performed in the present study.

ACT has been shown to be highly effective against *P. falciparum* gametocytes except on the more mature stages of their development [3], [33]. This is supported by our finding of a rather low respective efficacy among patients harbouring gametocytes at baseline. MB-based combinations not only cleared all pre-existing gametocytes but appeared to have a stronger effect than AQ-AS on the emergence of young gametocytes following treatment. This points to the possibility of a synergistic use of MB and artemisinin derivatives against gametocytes. Unfortunately, the small sample size of the present study did not allow probing this hypothesis.

The mechanism by which MB acts against gametocytes is obscure so far. Rapid elimination of asexual parasites (including fractions committed to gametocytogenesis) by MB-based combination treatment may be involved in preventing subsequent gametocytaemia. However, MB-AQ exhibited superior gametocytocidal effects but slower clearance of asexual parasites as compared to AQ-AS [25]. Interestingly, the remarkable effect of low doses of a MB derivative, plasmoquine, on *P. falciparum* gametocytes has long been known. In Sudanese children in the early 1930s, plasmoquine given prophylactically at a dosage of 0.02 mg/kg twice weekly, i.e. at a fraction of the therapeutical dose, almost completely prevented the occurrence of gametocytes [34]. Similarly, plasmoquine chemoprophylaxis taken twice a week yielded a substantial reduction of infected mosquitos in rubber plantation camps in Liberia in 1931 which was attributed to the drug's impact on gametocyte carriage [35]. Eventually, the potential of plasmoquine to interrupt transmission was recognized and recommended for this purpose [36]. The successor of plasmoquine, and structurally closely related, is primaquine. Primaquine not only is the drug of choice to eliminate hypnozoites in vivax or ovale malaria. Also, in experimental infections, the drug distinctly reduced the number of circulating gametocytes and sterilised those remaining [37]. In a recent trial in Tanzania, single dose primaquine given on the third day of treatment with sulfadoxine-pyrimethamine (SP) *plus* AS reduced gametocyte carriage by more than 80% as compared to SP-AS alone [32]. However, asexual blood stages of *P. falciparum* are not affected by therapeutical doses of primaquine, and a reduced gametocytocidal activity has been reported from India [38], [39]. It might be premature to consider MB as an alternative to primaquine as an adjunct to *P. falciparum* treatment, intended to block post-treatment transmission. Yet, MB shows promising features in this regard including strong activity against asexual parasites, developing and mature gametocytes, as well as synergism with artemisinine derivatives [25], [33]. Possibly, and in analogy to plasmoquine, MB exerts its gametocytocidal effects at dosages below those used in treatment which might be beneficial in terms of dosing and tolerability. Taken together, these features warrant further investigations of MB as a partner compound in ACT and non-ACT combinations as well as in blocking transmission. In this regard, studies on the infectivity of gametocytes from MB-treated individuals are needed.

In summary, this study provides evidence that suggests a high efficacy of MB-based combination therapy against *P. falciparum* gametocytes. A combination of MB with an artemisinin derivate may thus maximise the gametocytocidal effects of the latter. These findings add to already existing *in vitro* and *in vivo* evidence of a synergy between MB and artemisinins [25], [40]. If such findings would be confirmed in larger studies and considering the impending advent of artemisinin resistance [16]–[18], including MB in ACT schemes would be an interesting option to improve treatment efficacy, prevent the development of resistance, and to reduce the transmission of (resistant) *P. falciparum* parasites.

## Supporting Information

Checklist S1 CONSORT Checklist
(0.06 MB DOC)

Protocol S1 Trial Protocol
(0.20 MB DOC)
